# Identification of Primary Medication Concerns Regarding Thyroid Hormone Replacement Therapy From Online Patient Medication Reviews: Text Mining of Social Network Data

**DOI:** 10.2196/11085

**Published:** 2018-10-24

**Authors:** So Hyun Park, Song Hee Hong

**Affiliations:** 1 Research Institute of Pharmaceutical Science College of Pharmacy Seoul National University Seoul Republic Of Korea; 2 Korea Institute of Drug Safety and Risk Management Drug Safety Information Office of Adverse Drug Reaction Relief Anyang Republic Of Korea

**Keywords:** medication counseling, social network data, primary medication concerns, satisfaction with levothyroxine treatment

## Abstract

**Background:**

Patients with hypothyroidism report poor health-related quality of life despite having undergone thyroid hormone replacement therapy (THRT). Understanding patient concerns regarding levothyroxine can help improve the treatment outcomes of THRT.

**Objective:**

This study aimed to (1) identify the distinctive themes in patient concerns regarding THRT, (2) determine whether patients have unique primary medication concerns specific to their demographics, and (3) determine the predictability of primary medication concerns on patient treatment satisfaction.

**Methods:**

We collected patient reviews from WebMD in the United States (1037 reviews about generic levothyroxine and 1075 reviews about the brand version) posted between September 1, 2007, and January 30, 2017. We used natural language processing to identify the themes of medication concerns. Multiple regression analyses were conducted in order to examine the predictability of the primary medication concerns on patient treatment satisfaction.

**Results:**

Natural language processing of the patient reviews of levothyroxine posted on a social networking site produced 6 distinctive themes of patient medication concerns related to levothyroxine treatment: how to take the drug, treatment initiation, dose adjustment, symptoms of pain, generic substitutability, and appearance. Patients had different primary medication concerns unique to their gender, age, and treatment duration. Furthermore, treatment satisfaction on levothyroxine depended on what primary medication concerns the patient had.

**Conclusions:**

Natural language processing of text content available on social media could identify different themes of patient medication concerns that can be validated in future studies to inform the design of tailored medication counseling for improved patient treatment satisfaction.

## Introduction

Patients with hypothyroidism are treated with the most frequently prescribed drug in the United States, levothyroxine [1], but report poor quality of life despite the treatment [2-4]. Poor quality of life while receiving the treatment possibly reflects the different views that clinicians and patients hold toward the treatment success of thyroid hormone replacement therapy (THRT). Clinicians judge the treatment success based on a restored reference range of thyroid hormone in the blood. On the other hand, patients feel that the treatment is successful when they are free of symptoms such as fatigue, depression, and muscle cramps that are specific to hypothyroidism. Historically, the patient view has not been given adequate attention. Now, scientific evidence has grown for the health care community to embrace the patient-centric paradigm. Specifically for THRT, endocrinologists have come to accept the patient view following a series of studies that reported that some patients do not in fact get better on THRT, despite having a reference range of the thyroid hormone in the bloodstream. For this reason, European and American thyroid professional associations now acknowledge that patients who do not thrive on levothyroxine (T4) alone may try a combination therapy of T4 and T3 (liothyronine) [5,6]. Armour, a desiccated natural thyroid hormone, offers such a combination. A synthetic version also exists with a 4:1 mix of T4 and T3 and a more consistent composition than Armour [6,7]. However, routine use of these combinations is not recommended [8].

Studies pertaining to the patient view have mostly relied on questionnaire-based surveys that employ structured measurements of patient-reported outcomes [9-11]. Perhaps a better way to understand the patient view would be to explore an alternative source of data. Patients have begun to report their health care experiences on social networks [11]. As these reports come directly from patients with no filtering in between, they have the potential to genuinely represent patient experiences. Furthermore, they continuously accumulate on a large scale every day. A Pew Research study in 2012 reported that 61% of adults have read someone else’s health experience on a social network [12]. Many health websites such as WebMD and Ask-a-Patient provide open forums for patients to post ratings and reviews regarding their medication experience [13]. These online communities have become a valuable source of information for the study of patient medication experience [14].

Recent advancement in natural language processing (NLP) has enabled researchers to analyze unstructured text data posted by patients on social networks. NLP of text content on health-related social media has helped identify myriad health topics across a wide range of demographics [15]. Specifically in the area of THRT, NLP has been used to compare the patient ratings of levothyroxine and Armour along with their respective performance potentials [13]; the authors of the comparative study had recognized that patients were dissatisfied with levothyroxine and thus aimed to document the potential superiority of Armour [13]. However, before steering patients away from levothyroxine, a study needs to be conducted fully documenting the concerns of patients being treated with levothyroxine. Our study aimed to identify the distinctive themes of patient concerns regarding levothyroxine treatment based on an alternative data source in the form of a social network to inform the importance of patient-tailored medication counseling for THRT.

The specific aims of this study were to (1) identify the distinctive themes of patient concerns regarding THRT; (2) determine whether patients have unique primary medication concerns specific to their age, gender, and treatment duration; and (3) determine the predictability of primary medication concern on patient satisfaction with THRT, controlling for age, gender, treatment duration, and rating year. The results of this study can inform the design of a patient-centric medication therapy management that accounts for concerns unique to individual patients.

## Methods

### Study Design and Settings

Data for this study came from patient reviews and ratings of levothyroxine posted on a social network site (WebMD) in the United States between September 1, 2007, and January 30, 2017. WebMD runs a social network service that allows patients to browse patient reviews of prescription drugs based on their medication experience and to post their own reviews. Patients wishing to post their own reviews choose ratings on a scale of 1 to 5 stars for effectiveness, ease of use, and drug satisfaction and choose a reason for taking the drug. Patients can also answer questions relating to age, sex, and treatment duration. In addition, they can provide an open-ended comment. Finally, patients are required to enter the letters of a Captcha-like picture visible on the screen to prevent illegitimate use by automated programs and indicate that they agree to abide by the WebMD terms and conditions and privacy policy.

### Text Mining

Text mining required two preprocesses for building text corpora from the patient reviews. The first preprocess was cleaning, through which stop-words such as articles, numbers, punctuations, and demonstrative pronouns were removed from the text. Additionally frequently appearing but irrelevant words such as “take,” “im,” “cant,” “dont,” “thyroid,” “taken,” “t,” “now,” “takeing,” “takes,” “taking,” “age,” “ive,” “also,” “almost,” “els,” “else,” “far,” and “since” were also removed. The second preprocess was stemming, which reduced the words to their “stems.” Finally, a document-term matrix consisting of words along with their frequencies appearing in all the reviews was constructed. The frequency of each word was calculated from the term frequency-inverse document frequency [16,17]. For conducting the data preprocesses and analyses, open source R version 3.3.2 (The R Foundation) was used.

NLP was used to automate the task of information retrieval, analysis, and prediction inherent in languages used in patient reviews. Specifically, basic NLP methods such as tokenization, stop words, and stemming were used to process the content of patient reviews using the R software.

Latent Dirichlet allocation (LDA) was used to automatically discover hidden topics from a set of patient reviews, each of which contained a bag of words. The algorithm treated each review as a mixture of several topics and each topic as a distribution of words. By understanding topic and word distributions among the patient reviews, hidden information in the text could be found automatically [18]. For the LDA topic modeling, the numbers to be set of initial samples to be discarded, sampling iterations, and topics were 4000, 2000, and 6, respectively. The LDA package of the open source R was used as the analysis tool.

### Statistical Analysis

The frequency of each theme of patient medication concern was computed for each review and then summed for all reviews. The chi-square test was performed to test whether the distribution of themes of patient medication concerns varied with age, sex, and treatment duration. The percent distribution of themes of medication concerns was also computed for each subgroup of age, gender, and treatment duration along with their standard deviations. Statistical significance was tested at an alpha of .05. To determine the predictability of primary medication concern on treatment satisfaction, multiple linear regression analysis was conducted, controlling for age, sex, treatment duration, and the year the rating was posted.

## Results

### Description of Patient Medication Reviews

The total number of patient medication reviews and ratings posted on WebMD collected for this study was 2112, which included 1037 reviews of levothyroxine and 1075 reviews of its brand version, Synthroid. After eliminating those that had no comments, the number decreased to 1768 (Table 1). While the reviews were mostly posted by patients themselves (1694/1768, 95.81%), in rare cases (27/1768, 1.53%) they were posted by caregivers. Interestingly, almost half of the reviews (772/1768, 43.67%) were posted by those who had taken the drug for less than a year. Regarding the reason for taking the drug, underactive thyroid (1300/1768, 73.53%) was the most common, followed by thyroid cancer (115/1768, 6.50%). Females (1485/1768, 83.99%) exceeded males (214/1768, 12.1%) with respect to posting of reviews. As for the age of the reviewers, the 45- to 64-year-old group was the most dominant (863/1768, 48.81%). The reviews were posted between 2007 and 2017 with the largest number (351/1768, 19.85%) posted in 2009, followed by 2010 (321/1768, 18.16%) and 2011 (234/1768, 13.24%). Since 2011, posts had a steadily declining trend.

### Identification of Distinctive Themes of Patient Medication Concerns

The LDA topic modeling of the patient reviews produced 6 distinctive topics, each of which captured a hidden theme of patient medication concern (Table 2). Each hidden theme identified was given a name representing a certain aspect of medication treatment based on the top 20 most frequently appearing words [19]. A gallery of word clouds lists a complete set of words contained in each theme (Multimedia Appendix 1). The theme of patient medication concern about how to take the drug was named based on the keywords “work,” “time,” “hour,” “dose,” “water,” and “morning.” This concern had the least frequency share (300/2194, 13.67%); that is, it appeared least frequently out of the 6 themes of medication concerns. Likewise, the remaining 5 themes of patient medication concerns (treatment initiation, dose adjustment, symptoms of pain, generic substitutability, and appearance) were identified from scouting the list of top 20 keywords. The appearance theme of patient medication concern was named after scouting the keywords of “weight gain,” “hair loss,” and “dried skin.” The theme of appearance arose most frequently (436/2194, 19.87%), followed by dose adjustment (396/2194, 18.05%) and symptoms of pain (379/2194, 17.27%). The dose adjustment theme had keywords like “mcg,” “dosage,” “increase,” “change,” and “removal” in the list of top 20 words. The symptoms of pain theme had words like “severe,” “bad,” “pain,” “symptom,” “headache,” “heart,” “bodies,” “muscle,” “fatigue,” and “leg” in the list. Treatment initiation had keywords like “feel,” “just,” “better,” “start,” “sleep,” “first,” and “time” in the list. Generic substitutability had keywords like “synthroid,” “generic,” brand,” “blood,” and “test.”

### Uniqueness of Primary Medication Concerns Among Different Patients

**Figure 1:**
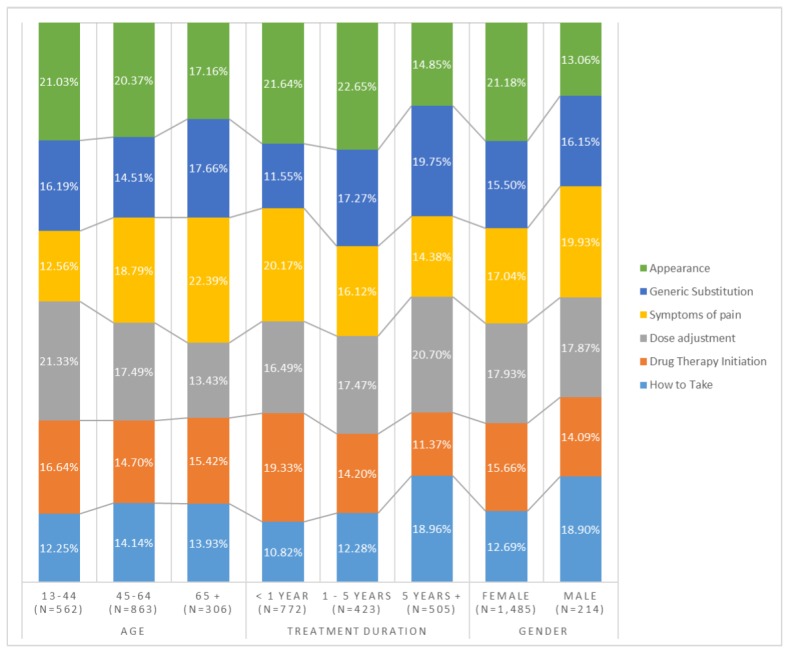
Primary medication concerns unique to patients of different backgrounds.

It was of interest to determine whether patients had unique medication concerns regarding levothyroxine treatment specific to their gender, age, and treatment duration. First, for gender, female patients mentioned “appearance” (mean 21.18% [SD 0.41]) as the most frequent concern (or primary medication concern) and how to take the drug (mean 12.69% [SD 0.33]) as the least frequent concern (Figure 1). However, male patients were least concerned about appearance (mean 13.06% [SD 0.34]) and most concerned about symptoms of pain (mean 19.93% [SD 0.40]).

Patient medication concerns regarding dose adjustment, symptoms of pain, and appearance were quite different according to age. The youngest (13 to 44 years) were the most concerned about dose adjustment (mean 21.33% [SD 0.41]) and appearance (mean 21.03% [SD 0.41]) while the oldest (65 years and older) were most concerned about symptoms of pain (mean 22.39% [SD 0.42]). Patient medication concerns were also quite different with treatment duration. Patients on levothyroxine less than a year were more concerned about appearance (mean 21.64% [SD 0.41]) and drug therapy initiation (mean 19.33% [SD 0.39]) while patients on levothyroxine 5 years or longer were more concerned about how to take the drug (mean 18.96% [SD 0.39]), dose adjustment (mean 20.70% [SD 0.41]), and generic substitutability (mean 19.75% [SD 0.40]).

**Table 1:** Characteristics of patient medication review writers.

Demographics		Total (n=1768), n (%)
**Gender**		
	Male	214 (12.10)
	Female	1485 (83.99)
	N/A	69 (3.90)
**Age, years**		
	13-44	562 (31.79)
	45-64	863 (48.81)
	65 and over	306 (17.31)
	N/A	37 (2.09)
**Treatment duration, years**		
	Less than 1	772 (43.67)
	1-5	423 (23.93)
	More than 5	505 (28.56)
	N/A	68 (3.85)
**Reasons of taking levothyroxine**		
	Progressive disease of the thyroid gland	56 (3.17)
	Additional treatment for thyroid cancer	1 (0.06)
	Decreased thyroid function existing from birth	20 (1.13)
	Enlarged thyroid gland	42 (2.38)
	Myxedema coma	1 (0.06)
	Serious decrease in thyroid function	81 (4.58)
	Thyroid cancer	115 (6.50)
	Underactive thyroid	1300 (73.53)
	Other	152 (8.60)
**Reviewer type**		
	Caregiver	27 (1.53)
	Patient	1694 (95.81)
	N/A	42 (2.66)
**Year**		
	2007	73 (4.13)
	2008	210 (11.88)
	2009	351 (19.85)
	2010	321 (18.16)
	2011	234 (13.24)
	2012	178 (10.07)
	2013	197 (11.14)
	2014	107 (6.05)
	2015	39 (2.21)
	2016	54 (3.05)
	2017	4 (0.23)

**Table 2:** Identification of 6 distinctive themes of patient medication concerns about levothyroxine from patient reviews.

Theme name	Top 20 words	Frequency (n=2194^a^), n (%)
How to take the drug	work, will, can, dose, help, time, eat, need, use, life, one, well, hour, good, know, thing, morning, anything, people, levothyroxine	300 (13.67)
Treatment initiation	feel, day, like, get, just, better, week, start, much, medicine, want, sleep, make, pill, first, night, know, think, time, keep	343 (15.63)
Dose adjustment	medicaid, year, mcg, effect, side, dosage, increase, change, noticeable, differ, diagnose, hypothyroidism, hope, little, seem, removal, two, disease, old, great	396 (18.05)
Symptoms of pain	med, drug, problem, pain, even, stop, heart, low, cause, severe, bodies, bad, fatigue, headaches, anyone, worse, muscle, symptom, leg, look	379 (17.27)
Generic substitutability	synthroid, doctor, back, level, blood, test, put, normal, generic, never, trial, went, right, TSH, symptom, felt, told, year, said, brand	340 (15.50)
Appearance	weight, gain, month, hair, still, energized, tired, loss, start, see, lbs, time, lost, depressant, lose, always, skin, pound, dried, fall	436 (19.87)

^a^Total number of primary themes represented in each review. This is greater than the total number of reviews (1768) because some reviews had more than one theme tied for the primary concern.

**Table 3:** Multiple regression analysis on factors affecting satisfaction score.

Characteristics		Coefficient (β)	Standard error	*P* value
Intercept		3.11	0.12	<.001
**Primary medication concern**				
	Appearance (reference)	—	—	—
	How to take the drug	0.68	0.12	<.001
	Treatment initiation	–0.03	0.11	.81
	Dose adjustment	0.44	0.11	<.001
	Symptoms of pain	–0.29	0.11	.01
	Generic substitutability	0.11	0.11	.32
**Treatment duration, years**				
	More than 5 (reference)	—	—	—
	Less than 1	–0.76	0.08	<.001
	1-5	–0.38	0.10	<.001
**Age, years**				
	45-64 (reference)	—	—	—
	13-44	0.12	0.08	.14
	65 and over	0.03	0.10	.76
**Gender**				
	Female (reference)	—	—	—
	Male	0.24	0.11	.03
Rating year		–0.04	0.02	.01

### Dependency of Treatment Satisfaction on Primary Medication Concerns

The last aim of this study was to determine whether patient treatment satisfaction with THRT depends on what primary medication concerns they have, controlling for age, gender, and treatment duration. For this aim, each review’s star rating of levothyroxine was treated as an interval scale rating. The 6 themes of patient medication concerns were dichotomized to indicate whether each medication concern was primary for the patient. As compared to the primary medication concern regarding appearance, the primary medication concern regarding how to take the drug resulted in a significantly better treatment satisfaction, up to as much as 0.68, with gender, age, treatment duration, and rating year being constant (Table 3). In other words, when patients had how to take the drug as their primary medication concern, their treatment satisfaction with THRT was 0.68 points higher out of 5 stars compared to those patients who had appearance as their primary medication concern. Treatment satisfaction also significantly increased (by 0.44 points) for the primary medication concern dose adjustment but reduced by 0.29 points (*P*=.01) for the primary medication concern symptoms of pain compared to the reference primary medication concern of appearance. Interestingly, treatment initiation and generic substitution did not have a significant impact on the treatment satisfaction with THRT relative to appearance.

Regarding the potential confounders controlled in the regression analysis, age did not result in any significant explanation of treatment satisfaction with THRT. For gender, male patients reported a significantly higher (β=0.24, *P*=.03) treatment satisfaction than female patients. For treatment duration, the longer the treatment duration, the higher the treatment satisfaction. In other words, as compared to the reviews written by patients who had taken levothyroxine for more than 5 years, the reviews written by patients who had taken the drug for shorter durations had significantly lower levels of treatment satisfaction.

Finally, multiple regression had the variable of rating year as an explanatory variable. The rating year represents the year the review was posted. For reviews posted in more recent years, the treatment satisfaction was slightly but significantly lower than the previous years. A 1-year increase in the rating year was associated with a decline of 0.04 points (*P*=.01) in the treatment satisfaction.

## Discussion

### Principal Findings

Above all, the NLP of patient medication reviews posted on a social network identified 6 distinctive themes of patient medication concerns on THRT: how to take the drug, treatment initiation, dose adjustment, symptoms of pain, generic substitutability, and appearance. Those 6 themes seem to cover all areas of medication needs of patients on THRT. The patient medication concerns on how to take the drug and dose adjustment quite well reflect the tough challenges associated with the task of taking levothyroxine appropriately. Levothyroxine requires scrupulous planning regarding when to take it. Many factors such as food, medications, and health conditions hamper the ability to maintain a desired range of thyroxine in the bloodstream [20]. It is critical to maintain the desired range because of the narrow therapeutic index and low absorption rate [21,22]. This study successfully identified those challenges into primary medication concerns how to take the drug and dose adjustment.

Likewise, the themes of symptoms of pain and appearance are well documented in the literature. Patients on THRT are known to complain about various symptoms of pain such as fatigue; loss of sleep; heart issues; headache; and muscle, leg, and body pain [2-4]. For this reason, a patient-reported outcomes instrument, Thyroid-Dependent Quality of Life, was developed to measure those symptoms of pain using several items on bodily discomfort [23]. Further, the most frequently mentioned medication concern was on appearance. A study reports that patients on THRT are often dissatisfied with levothyroxine treatment because of weight gain, hair loss, and dry skin [24].

Last, patients were quite frequently (15.5%) concerned about generic substitutability. Considering the price difference between brands and generic versions, patients would certainly rather use a generic version. To help ease patient concerns on generic substitutability, in 1997 the US Food and Drug Administration made the equivalence criteria stricter, from a range of 90% to 110% before to 95% to 105% after [25-27]. However, the issue of generic substitutability is still controversial. The American Thyroid Association reports that in a small fraction of patients higher rates of adverse events were associated with changes in levothyroxine preparations [6]. Furthermore, a reformulation of Merck’s Lévothyrox with different excipients to reduce variability in dosing from 90% to 110% to 95% to 105% led to a huge controversy in France in 2017 (thousands of patients made complaints about adverse effects after taking the reformulated version [28]).

Documenting the uniqueness of primary medication concerns among different patients would emphasize the importance of tailored medication counseling. This study found that primary medication concerns were unique to patients of different genders, ages, and treatment durations. Female patients had appearance as their primary medication concern while male patients mentioned how to take the drug quite often. While it is apparent that females are quite attentive to appearance, it raises the question why males are more concerned about how to take the drug. Taking levothyroxine correctly requires careful planning regarding foods and time of administration. Perhaps females are accustomed to planning these things for family members, and thus, are more confident about taking the medication correctly than males.

This study also found that the youngest patients (aged 13 to 44 years) were very concerned about dose adjustment (21.33%) and appearance (21.03%) while the oldest (aged 65 years and older) were most concerned about symptoms of pain (22.39%). It is understandable that younger people were concerned about appearance. However, it requires some explanation why younger people were concerned about dose adjustment. Younger people must have just begun the levothyroxine treatment, and thus it would take longer for them to get the dose adjusted. On the other hand, older people were most concerned about the symptoms of pain because they were already suffering from many symptoms of pain resulting from aging. Patients with hypothyroidism would certainly experience symptoms of hypothyroidism unique to their age [22].

As the treatment duration became longer, patients were less concerned about appearance and treatment initiation and became more concerned about how to take the drug, dose adjustment, and generic substitution. Patients who had taken the drug for quite a while may have realized that it was more challenging to control hypothyroidism with levothyroxine. Once they realized that it is challenging, they would be more concerned about how to take the drug and dose adjustment. They would also be concerned about generic substitutability because a longer treatment duration makes it economically burdensome to take the expensive brand name drug.

Last, patient satisfaction with levothyroxine treatment depended on their primary medication concern. Patients who had the primary medication concerns of how to take the drug and dose adjustment were more satisfied with the levothyroxine medication therapy (up 0.68 and 0.44, respectively) compared to those whose primary medication concern was appearance. Evidently, patients who were more concerned about how to get the THRT right would have achieved better treatment outcomes. On the other hand, patients who had a primary medication concern of symptoms of pain were less satisfied with THRT (down 0.29 points) than the referents. Naturally, symptoms of pain would have negatively affected treatment satisfaction.

One of the interesting study findings was that the longer the treatment duration, the higher the treatment satisfaction. Patients who had taken the drug longer were more likely to have known how to take the drug correctly with the right dose and thus to have achieved better treatment outcomes. Moreover, males were more satisfied with the treatment than females. Because males were more concerned about how to take the drug but less concerned about appearance than females, they were more likely to achieve treatment success, which leads to higher satisfaction scores.

### Practice Implication

Patients with hypothyroidism had unique primary medication concerns depending on their identity. In order to improve their treatment outcomes, it is necessary to provide tailored medication counseling, specifically focusing on their primary medication concerns such as how to take the drug, dose adjustment, symptoms of pain, and appearance.

### Limitations

First, the LDA algorithm used in this research is a method of automatically discovering topics or themes by using statistical principles. Therefore, although LDA is advantageous in extracting hidden themes, the scientific qualities of the themes should be further validated. Furthermore, the researchers’ subjectivity might have played an important role in the process of extracting hidden themes. For example, this study set the number of themes to be extracted to 6 based on its manageability. However, research has yet to come up with an easy way to choose an optimal number of themes [29]. Second, there may be a bias that arises from using online reviews from social media. Considering that people are more likely to report negative views than positive ones, the patient reviews used for this study may have come from patients dissatisfied with levothyroxine. Although negative views could better reflect patient concerns, they do not represent all views on levothyroxine. It is also important to note that this study used patient reviews from only one social media platform. Data collected from multiple social media platforms would have increased representativeness. Finally, the reported themes of patient medication concerns on levothyroxine treatment may not be valid because they were based on unstructured text data directly reported from patients. While those text data have their own merits, the reported themes need to be tested for their validity in future studies.

### Conclusion

NLP of the patient reviews of levothyroxine posted on a social network produced 6 distinctive themes of patient medication concerns that covered all areas of levothyroxine treatment. Patients had unique primary medication concerns specific to their gender, age, and treatment duration. Furthermore, patients’ treatment satisfaction on levothyroxine varied depending on what primary medication conditions individual patients had. These results inform the design of patient-tailored medication counseling to accommodate unique primary medication concerns of individual patients [30].

## Appendix

Multimedia Appendix 1 Word clouds of primary medication concerns.

## Abbreviations

THRT: thyroid hormone replacement therapy
NLP: natural language processing
LDA: latent Dirichlet allocation
