# Supplementary figures and images for: Identification of Primary Medication Concerns Regarding Thyroid Hormone Replacement Therapy From Online Patient Medication Reviews: Text Mining of Social Network Data
Source: J Med Internet Res. 2018 Oct 24;20(10):e11085. doi: 10.2196/11085 (PMC6231751; doi:10.2196/11085)

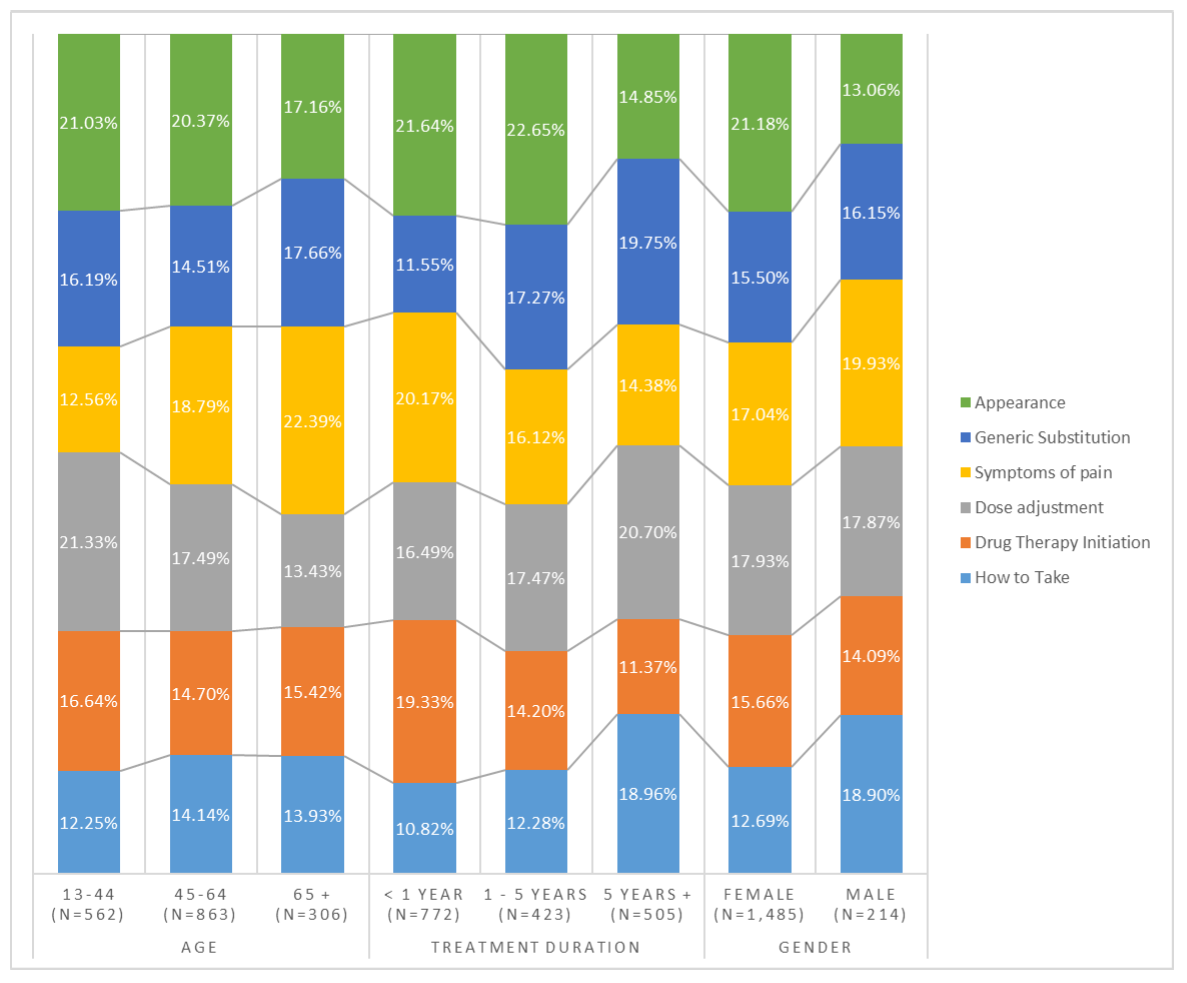

Supplement: Multimedia Appendix 1 [file jmir_v20i10e11085_fig1.png]
